# Longitudinal increase in albumin–bilirubin score is associated with non-malignancy-related mortality and quality of life in patients with liver cirrhosis

**DOI:** 10.1371/journal.pone.0263464

**Published:** 2022-02-03

**Authors:** Akira Sakamaki, Masaaki Takamura, Norihiro Sakai, Yusuke Watanabe, Yoshihisa Arao, Naruhiro Kimura, Toru Setsu, Hiroyuki Abe, Takeshi Yokoo, Hiroteru Kamimura, Shunsuke Tsubata, Nobuo Waguri, Toru Ishikawa, Hirokazu Kawai, Soichi Sugitani, Tomomi Sato, Kazuhiro Funakoshi, Masashi Watanabe, Kentarou Igarashi, Kenya Kamimura, Atsunori Tsuchiya, Yutaka Aoyagi, Shuji Terai

**Affiliations:** 1 Division of Gastroenterology and Hepatology, Graduate School of Medical and Dental Sciences, Niigata University, Niigata, Japan; 2 Division of Gastroenterology and Hepatology, JA Niigata Kouseiren Nagaoka Chuo General Hospital, Niigata, Japan; 3 Department of Preemptive Medicine for Digestive Diseases and Healthy Active Life, School of Medicine, Niigata University, Niigata, Japan; 4 Department of Gastroenterology and Hepatology, Tachikawa General Hospital, Niigata, Japan; 5 Department Gastroenterology and Hepatology, Niigata City General Hospital, Niigata, Japan; 6 Division of Gastroenterology and Hepatology, Saiseikai Niigata Hospital, Niigata, Japan; 7 Department of Internal Medicine, Niigata Prefectural Shibata Hospital, Niigata, Japan; 8 Department of Internal Medicine, Murakami General Hospital, Niigata, Japan; 9 Department of Internal Medicine, Joetsu General Hospital, Niigata, Japan; 10 Division of Gastroenterology and Hepatology, Niigata Prefectural Hospital, Niigata, Japan; 11 Division of Gastroenterology and Hepatology, Mitsuke City Hospital, Niigata, Japan; 12 Department of General Medicine, School of Medicine, Niigata University, Niigata, Japan; 13 Division of Gastroenterology and Hepatology, JA Niigata Medical Center, Niigata, Japan; Nihon University School of Medicine, JAPAN

## Abstract

Due to the developments in the treatment for hepatitis, it is possible to prevent the progression of liver fibrosis and improve patients’ prognosis even if it has already led to liver cirrhosis (LC). Consequently, a two-step study was conducted. To begin with, a retrospective study was conducted to identify the potential predictors of non-malignancy-related mortality from LC. Then, we prospectively analyzed the validity of these parameters as well as their association with patients’ quality of life. In the retrospective study, 89 cases were included, and the multivariate Cox regression analysis indicated that age (P = 0.012), model for end-stage liver disease (MELD) score (P = 0.012), and annual rate of change of the albumin–bilirubin (ALBI) score (P < 0.001) were significantly associated with LC prognosis. In the prospective study, 70 patients were included, and the patients were divided into cirrhosis progression and non-progression groups. The univariate logistic regression analysis indicated the serum procollagen type III N-terminal peptide level (P = 0.040) and MELD score (P = 0.010) were significantly associated with the annual rate of change of the ALBI score. Furthermore, the mean Chronic Liver Disease Questionnaire score worsened from 5.3 to 4.9 in the cirrhosis progression group (P = 0.034). In conclusion, a longitudinal increase in the ALBI score is closely associated with non-malignancy-related mortality and quality of life.

## Introduction

Liver cirrhosis (LC) is the end stage of a final shared pathway in chronic damage with the irreversible fibrosis. D’Amico et al. reported that the 5-year survival rates of LC are approximately 75% and 25% for compensated and decompensated, respectively, and the annual rate of progression from compensated to decompensated LC is 5%–7% [1]. In Japan, Maesaka et al. reported that the 2-year survival rate for decompensated LC due to the hepatitis C virus is 64.8% [2]. There is no established treatment for decompensated LC aside from liver transplantation. In Japan, liver transplantation is a limited therapeutic option because of the severe shortage of donated organs [3, 4]. Therefore, it is important to preserve liver function to prevent decompensation that may result in a poor prognosis. Recent developments in the treatment of viral hepatitis, such as direct-acting antivirals for hepatitis C [5] and nucleoside/nucleotide analogs for hepatitis B [6], have made it possible to regulate these diseases.

Controlling the underlying liver damage can prevent the progression to liver fibrosis. In addition, it can improve the prognosis of patients [7, 8]. However, the “point of no return” exists, which is the progression of LC even after hepatitis has been regulated, as typified by hepatitis C [9, 10]. Several factors related to the prognosis of LC have been reported, such as the Child–Pugh grade [1], model for end-stage liver disease (MELD) score [1], renal impairment [11], liver stiffness (ultrasound-based transient elastography or magnetic resonance elastography) [12, 13], the combination of liver stiffness and MELD score [14], hepatic venous pressure gradient [15], sarcopenia [16], and albumin–bilirubin (ALBI) score [17]; however, these factors do not assess the course of progression.

In the management of LC, it is important to preserve the patient’s quality of life (QOL), as same as to improve the prognosis [18]. Therefore, a two-step multicenter study was performed in Niigata Prefecture. To begin with, a retrospective study was conducted to identify the potential predictors of death from non-malignant LC. Then, in the prospective study, we evaluated the validity of the parameters and investigated the relationship between the parameters and the QOL using the Chronic Liver Disease Questionnaire (CLDQ) score.

## Materials and methods

### Retrospective study design

**Fig 1:**
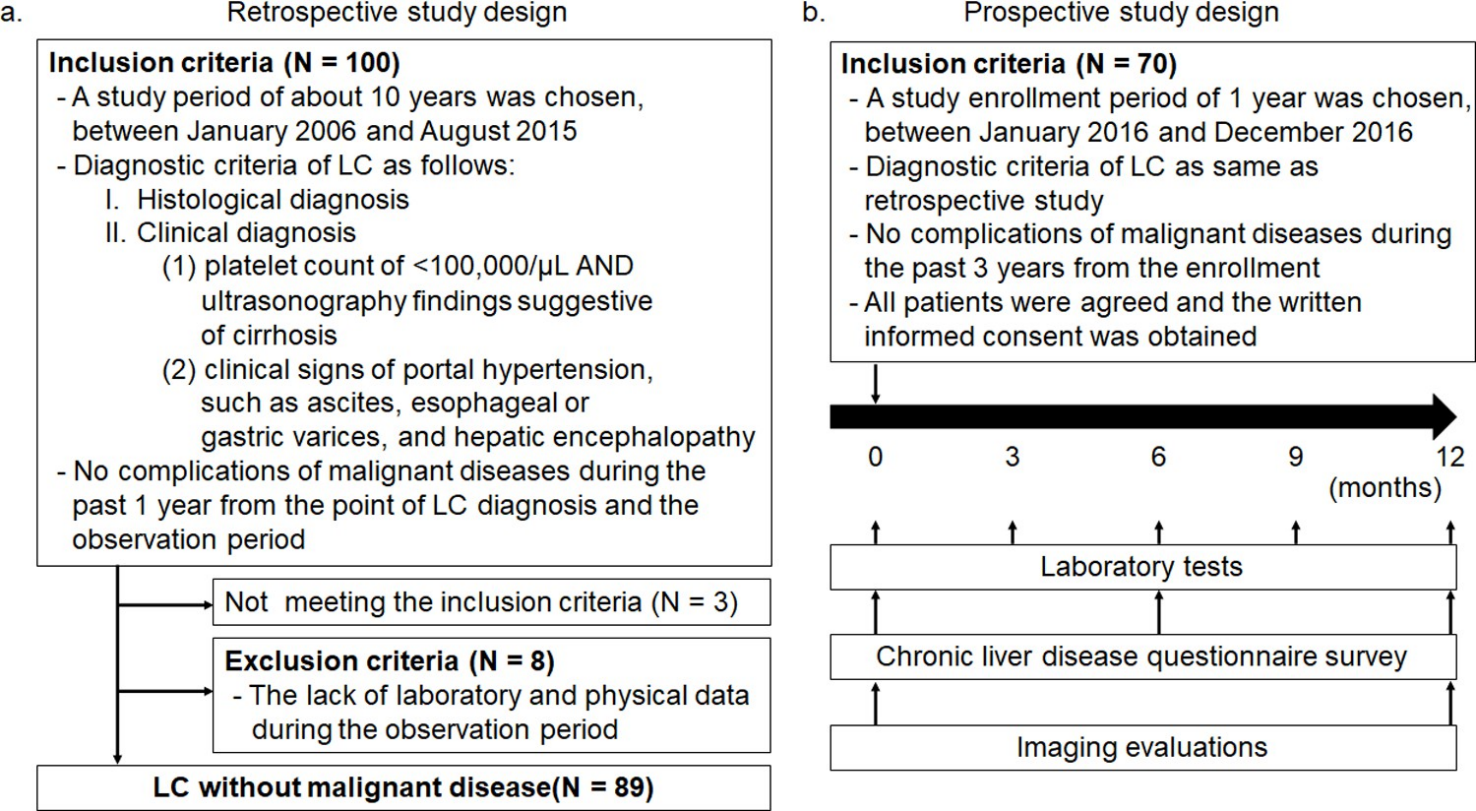
The entry procedure and study protocol. Retrospective study (A) and prospective study (B) protocols are shown. LC, liver cirrhosis.

This was a multicenter study conducted in Niigata Prefecture. The study was approved by the ethical review board of Niigata University (approval nos. 2015–2306 for the retrospective study and 2015–2307 for the prospective study). Anonymized data were obtained from all of the joint research facilities. We disclosed information for the retrospective study by the opt–out approach with the deliberation by the ethical review board. The authors will ensure that this study is conducted in accordance with the principles of the Declaration of Helsinki and with the ethical guidelines for medical and biological research involving human subjects in Japan. The retrospective study aimed to identify the potential predictor of death from non-malignancy-associated LC. In the retrospective analysis, data were collected from hospital medical records of patients diagnosed as having LC between January 2006 and August 2015. The diagnosis of LC was based on histological and clinical evidence. The clinical diagnosis of LC was based on a previous report [19] as follows: (1) platelet count of <100,000/μL and ultrasonography findings suggestive of cirrhosis and (2) clinical signs of portal hypertension, such as ascites, esophageal or gastric varices, and hepatic encephalopathy. The observation period was up to 5 years from the diagnosis of LC including dead or lost to follow-up cases within the observation period, and patients who had no complications of malignant diseases including hepatocellular carcinoma during the past 1 year from the point of LC diagnosis and the observation period were enrolled in the study. The overall target case number was set at 100, and each joint research facility enrolled a number of LC cases meeting the inclusion criteria according to the facility’s scale. Three cases were excluded for not meeting the inclusion criteria after data assessments at Niigata university. A total of 97 cases met the criteria, but 8 cases were excluded because of the missing data for the assessment of the LC statement. Finally, we analyzed 89 cases for the retrospective study. The entry procedure and retrospective study protocol are shown in Fig 1A.

Study endpoint was the death of cases unrelated to malignancies, including HCC, and no cases were treated by liver transplantation, and the study’s objective was to identify predictors of mortality. Based on the medical records, information on laboratory data, imaging examination, and clinical symptoms during the follow-up period was collected. Control of viral hepatitis was defined as continuous negative serum DNA level in hepatitis B or sustained virological response (SVR) in hepatitis C. In addition, information on the date of last observation and cause of death was collected in patients with an observation period of less than 5 years.

### Prospective study design

In the prospective analysis, patients who were diagnosed with LC between January 2016 and December 2016 and agreed to the study concept were included, and those who had complications of malignant diseases during the previous 3 years from the enrollment period were excluded. The observation period was up to 1 year from the study enrollment day including death or lost to follow-up cases within the observation period. A total of 70 cases were enrolled, and all of them were included in the analysis. Written informed consent was obtained from all patients for the prospective study enrollment. The laboratory tests and CLDQ survey were conducted every 3 and 6 months, respectively, and the imaging evaluations were performed at the start and end points of the study. The entry procedure and prospective study protocol are shown in Fig 1B. Study endpoint of prospective study was the annual change rate of the ALBI score, and outcomes were the CLDQ score.

### CLDQ

The CLDQ is a questionnaire consisting of 29 questions categorized into 6 domains (abdominal symptoms, fatigue, systemic symptoms, activity, emotional function, and worry) and published by Younossi et al. as a health-related QOL assessment method specific to liver diseases [18]. We used the Japanese version in the present study [20]. Each question of the CLDQ is classified into seven levels, from 1 (worst) to 7 (best), and the CLDQ score was assessed based on the average score of all the questions.

### Statistical analysis

Cox regression and Kaplan–Meier analyses were used to compare the prognosis. The linear approximation method was employed to calculate the annual rate of change of all the laboratory data in the observation period. The receiver operating characteristic (ROC) analysis was used to detect the involvement of the factors on the prognosis in the retrospective study, and the Youden index was utilized to decide the cutoff value of the ROC curve. The Kolmogorov–Smirnov test was used to assess the normality of the distribution of continuous variables. The Mann–Whitney U test, Fisher’s exact test, and logistic regression analysis were utilized to compare data of each cluster. The SPSS Statistics software (version 22.0; IBM, Armonk, NY, USA) was used to perform the Cox regression analysis, Kolmogorov–Smirnov test, Mann–Whitney U test, Fisher’s exact test, and logistic regression analysis, whereas the Prism software (version 8.30; GraphPad, La Jolla, CA, USA) was employed to perform the ROC and Kaplan–Meier analyses.

## Results

The patients’ backgrounds in the retrospective analysis are shown in Table 1. The study cohort included 42 male and 47 female patients (n = 89), with a mean age of 63.5 ± 11.6 years. Additionally, the median observation period was 60.0 months, 14 patients (15.7%) died during the study period, and the 5-year survival rate was 82.3%. Eight cases died from hepatic failure, two from systemic infection, two from bleeding, one from respiratory failure, and one from an unidentified cause. The univariate Cox regression analysis showed that age, serum alkaline phosphatase level, and MELD score at the start point of observation were the significant relevant factors in predicting the prognosis (P = 0.017, 0.039, and 0.021, respectively). In addition, the annual rate of change of the ALBI, MELD, and Child–Pugh scores was significantly related to the prognosis (P <0.001, <0.001, 0.004, respectively, Table 1).

**Table 1:** The patients’ backgrounds and cox regression analyses in the retrospective study.

	Mean ± SD	Univariable Cox regression		Multivariable Cox regression	
N = 89	or n (%)	Hazard ratio (95% CI)	P value	Hazard ratio (95% CI)	P value
**Age, years**	63.5 ± 11.6	**1.08 (1.01–1.15)**	**0.017** *	**1.09 (1.02–1.17)**	**0.012** *
**Gender**					
Males	42 (47.2)	1.13 (0.39–3.25)	0.825		
Females	47 (52.8)				
**Body mass index, kg/m** ^**2**^	24.6 ± 5.4	0.96 (0.86–1.08)	0.511		
**The etiology of liver cirrhosis**			0.767		
Hepatitis B virus	1 (1.1)				
Hepatitis C virus	34 (38.2)				
Alcohol	31 (34.8)				
Non-alcoholic steatohepatitis	23 (25.8)				
**Aspartate aminotransferase, U/L**	66 ± 50	1.01 (1.00–1.01)	0.102		
**Alanine aminotransferase, U/L**	38 ± 21	1.01 (0.99–1.03)	0.333		
**Alkaline Phosphatase, U/L**	394 ± 261	**1.00 (1.00–1.00)**	**0.039** *		
**Cholinesterase, U/L**	150 ± 67	1.00 (0.99–1.01)	0.491		
**Albumin, g/dL**	3.3 ± 0.6	0.60 (0.24–1.47)	0.263		
**Total bilirubin, mg/dL**	1.7 ± 1.3	1.15 (0.83–1.59)	0.393		
**Prothrombin time, %**	70 ± 18	0.98 (0.95–1.01)	0.179		
**C-reactive protein, mg/dL**	0.71 ± 1.71	0.95 (0.60–1.51)	0.825		
**Ammonia, μg/dL**	101 ± 53	1.00 (0.99–1.01)	0.454		
**Creatinine, mg/dL**	0.84 ± 0.47	1.83 (0.79–4.25)	0.158		
**Blood urea nitrogen, mg/dL**	17 ± 12	1.02 (0.98–1.05)	0.327		
**White blood cell count, x10** ^**3**^ **/μL**	4.9 ± 3.4	1.00 (1.00–1.00)	0.932		
**Platelet count, x10** ^**4**^ **/μL**	9.2 ± 4.7	0.94 (0.82–1.07)	0.342		
**Control of viral hepatitis (HBV-DNA negative or SVR)**	11 (31.4)	0.39 (0.05–3.21)	0.378		
**Child-Pugh score**	7 ± 2	1.22 (0.93–1.61)	0.158		
Child Pugh grade (A/B/C)	43 / 37 / 9	1.78 (0.84–3.78)	0.131		
**ALBI score**	-1.91 ± 0.61	1.80 (0.75–4.34)	0.188		
ALBI grade (1/2/3)	13 / 59 / 17	2.32 (0.92–5.83)	0.187		
**MELD score**	8.8 ± 4.3	**1.10 (1.01–1.18)**	**0.021** *	**1.14 (1.03–1.26)**	**0.012** *
**The annual rate of**					
**Platelet count, x10** ^**4**^ **/μL**	-0.13 ± 1.33	0.76 (0.52–1.10)	0.143		
**ALBI score**	0.07 ± 0.26	**65.54 (9.19–467.63)**	**<0.001** *	**38.36 (5.08–289.56)**	**<0.001** *
**MELD score**	0.91 ± 2.01	**1.32 (1.16–1.51)**	**<0.001** *		
**Child-Pugh score**	0.18 ± 0.60	**3.04 (1.44–6.42)**	**0.004** *		
**5-year survival rate**	82.3				

SD, standard deviation; CI, confidence interval; HBV, hepatitis B virus; SVR, sustained virological response; ALBI, Albumin-Bilirubin; MELD, model for end-stage liver disease

*: P value < 0.05.

**Fig 2:**
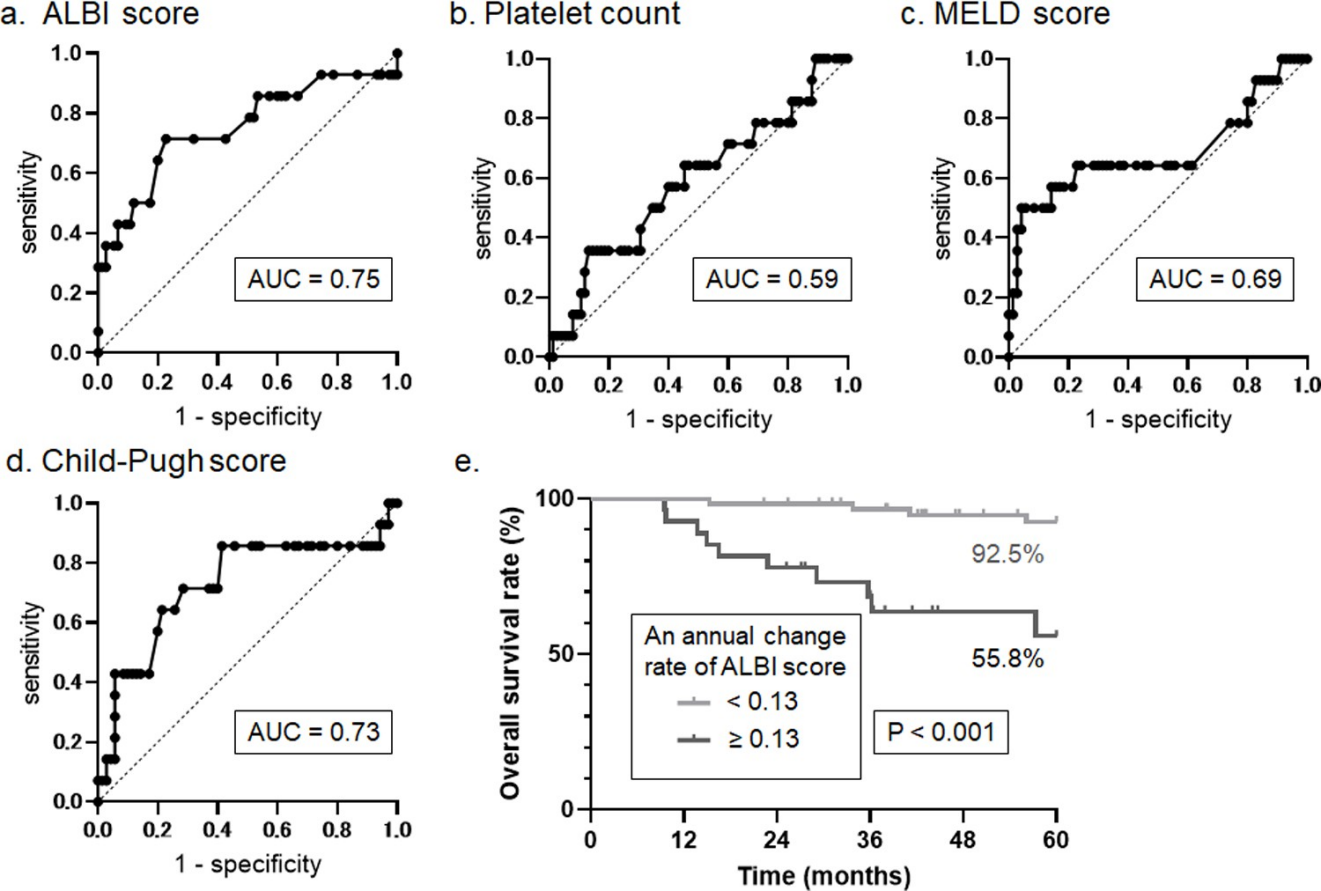
The annual rate of change of the liver function indices and their association with the prognosis.

As shown by the results of the Cox regression analysis, a worsening of the ALBI, MELD, and Child–Pugh scores was a more sensitive predictor of the non-malignancy-related mortality than the first point assessment of the liver function. Additionally, we investigated the relationship between the various annual rates of change of the liver function indices and the prognosis using ROC analyses. The area under the curve (AUC) of the analyses indicated that the annual rate of change of the ALBI score was mostly associated with the mortality in LC (with ALBI score of 0.75, platelet count of 0.59, MELD score of 0.69, Child–Pugh score of 0.73, Fig 2A–2D). However, the AUC of the annual rate of change of the ALBI had no significant difference from other indices since several components were duplicated, such as serum total bilirubin and prothrombin time (Table 2). Therefore, the annual rate of change in the MELD and Child-Pugh scores were excluded from the multivariate analysis.

**Table 2:** The receiver operating characteristic analysis in the retrospective study.

	Area under curve (95% CI)	Youden index	P value for the annual rate of ALBI score
**The annual rate of ALBI score**	**0.75 (0.59–0.91)**	**147.2**	–
Age	0.72 (0.55–0.88)	143.0	0.793
MELD score	0.70 (0.55–0.85)	138.8	0.624
The annual rate of Child-Pugh score	0.73 (0.56–0.89)	144.3	0.604
The annual rate of MELD score	0.69 (0.50–0.88)	145.7	0.375

ALBI, Albumin-Bilirubin; MELD, model for end-stage liver disease.

The AUC of the receiver operating characteristic analyses indicates that the annual change rate of the ALBI score is mostly associated with mortality (with ALBI score of 0.75, A; platelet count of 0.59, B; MELD score of 0.69, C; Child–Pugh score of 0.73, D). The Kaplan–Meier analysis indicates that the 5-year survival rates were 92.5% and 55.8% for patients who had an annual rate of change in the ALBI score of <0.13/year and ≥0.13/year, respectively (P < 0.001, E), according to the Youden index. AUC, area under the curve; ALBI, albumin–bilirubin; MELD, model for end-stage liver disease.

After that, we conducted the multivariate Cox analysis of the age, MELD score, and the annual rate of change in the ALBI score, and found that the results for all three factors were similar as the univariate analysis (Table 1). Serum alkaline phosphatase level was excluded in the multivariate Cox analysis because of the clinically meaningless (hazard ratio 1.00, 95% confidence interval 1.00–1.00). The annual rate of change in the ALBI score had the highest AUC with no statistical significance (Table 2) indicating that it was an independent sensitive predictor of non-malignancy-related mortality (P < 0.001, hazard ratio 38.36, 95% confidence interval 5.08–289.56, Table 1) as the same as age.

In addition, to evaluate the annual rate of change’s utility in a prospective study, Youden’s index was used to establish an optimal cutoff value. The Youden index of the annual rate of change of the ALBI score was 147.2, and the cutoff value was 0.13/year. The Kaplan–Meier analysis of the cutoff value indicated that the 5-year survival rates were 92.5% and 55.8% for patients who had an annual rate of change in the ALBI score of <0.13/year and ≥0.13/year, respectively (P < 0.001, Fig 2E). As the result of the retrospective study, the annual change of ALBI score is one of the sensitive predictors of non-malignancy-related mortality in patients with LC that speculated due to the progression of cirrhosis, therefore, an annual change rate in the ALBI score of ≥0.13/year defined the progression of LC in the study.

Followed by the retrospective study, we performed cluster analysis classified into two groups in the prospective study: the cirrhosis progression group and the cirrhosis non-progression group. The patients in the cirrhosis progression group had an ALBI score of ≥0.13/year or a fatal course during the observation period, whereas those in the cirrhosis non-progression group had an ALBI score of <0.13/year. Of the 70 cases, 7 cases were died, and 3 cases were out of follow during the observation period. The patients’ backgrounds in the prospective analysis are shown in Table 3. Based on the results of the comparison between the two groups, there was no significant difference in all of the laboratory data except for the serum cholinesterase and procollagen type III N-terminal peptide (P-III-NP) levels (P = 0.019, 0.024, respectively). Furthermore, in patients with LC due to viral hepatitis, the control rate of hepatitis was significantly different between groups (P = 0.033). To identify the factors that predict the elevation of ALBI score, univariate logistic regression analysis was performed. Parameters at the entry period were used for the analysis, including unusual parameters for the prediction of LC progression that not assessed in retrospective study, such as hyaluronic acid, type IV collagen 7s, P-III-NP, and WFA+-M2BP. The results showed that the serum P-III-NP level and MELD score were the significant relating factors to the elevation of ALBI score (P = 0.040, 0.010, respectively, Table 4). The control of viral hepatitis was also significant factor in univariate logistic regression analysis (P = 0.036), but it was excluded from multivariate logistic regression analysis because it was not assessed in LC patients without viral hepatitis. Therefore, the MELD score was only derived in the multivariate logistic regression analysis (P = 0.018, odds ratio 1.20, 95% confidence interval 1.03–1.40). Furthermore, univariate logistic regression analysis was limited in LC due to hepatitis C, SVR was the only significant factor (S1 Table).

**Table 3:** The patients’ backgrounds and cluster analysis in the prospective study.

Mean ± SD or n (%)	Non-progression group	Progression Group	Mann–Whitney U and Fisher’s exact tests
	N = 39	N = 31	P value
**Age, years**	64.2 ± 11.0	67.4 ± 10.6	0.216
**Gender**			
Males	22 (56.4)	21 (67.7)	0.459
Females	17 (43.6)	10 (32.3)	
**Body mass index, kg/m** ^**2**^	24.2 ± 4.4	26.3 ± 4.8	0.064
**The etiology of liver cirrhosis**			0.270
Hepatitis B virus	5 (12.8)	0 (0.0)	
Hepatitis C virus	9 (23.1)	10 (32.3)	
Alcoholic liver disease	15 (38.5)	15 (48.4)	
Non-alcoholic steatohepatitis	4 (10.3)	3 (9.7)	
Others	6 (15.4)	3 (9.7)	
**Aspartate aminotransferase, U/L**	42 ± 23	60 ± 55	0.112
**Alanine aminotransferase, U/L**	27 ± 19	36 ± 32	0.093
**Alkaline Phosphatase, U/L**	403 ± 196	460 ± 227	0.901
**Cholinesterase, U/L**	169 ± 66	139 ± 74	**0.019** *
**Albumin, g/dL**	3.4 ± 0.5	3.2 ± 0.7	0.062
**Total bilirubin, mg/dL**	1.4 ± 1.2	1.5 ± 1.3	0.284
**Prothrombin time, %**	74 ± 21	69 ± 22	0.338
**Ammonia, μg/dL**	69 ± 40	88 ± 61	0.263
**Creatinine, mg/dL**	0.78 ± 0.29	1.13 ± 1.26	0.123
**BTR**	4.45 ± 2.96	4.38 ± 2.67	0.836
**White blood cell count, x10** ^**3**^ **/μL**	5.0 ± 2.2	4.4 ± 2.2	0.225
**Lymphocyte count, x10** ^**3**^ **/μL**	1.3 ± 0.6	1.2 ± 0.8	0.202
**Platelet count, x10** ^**4**^ **/μL**	10.2 ± 4.7	10.0 ± 6.3	0.433
**Control of viral hepatitis (HBV-DNA negative or SVR)**	8 (57.1)	1 (10.0)	**0.033** *
**Hyaluronic acid, ng/mL**	434 ± 493	662 ± 693	0.092
**Type IV collagen 7s, ng/mL**	8.8 ± 4.8	9.3 ± 2.9	0.163
**P-III-NP, U/mL**	1.2 ± 0.5	1.5 ± 0.6	**0.024** *
**WFA** ^**+**^ **-M2BP, COI**	5.79 ± 3.92	7.41 ± 4.62	0.158
**Child-Pugh score**	7 ± 2	8 ± 2	0.055
Child Pugh grade (A/B/C)	23 / 11 / 5	11 / 15 / 5	0.121
**ALBI score**	-2.10 ± 0.57	-1.87 ± 0.64	0.070
ALBI grade (1/2/3)	7 / 26 / 6	4 / 20 / 7	0.639
**MELD score**	7.5 ± 2.3	9.9 ± 4.8	0.055
**Total CLDQ score Subscores**	5.1 ± 1.2	5.1 ± 1.0	0.976
Abdominal symptoms	5.6 ± 1.2	5.5 ± 1.3	1.000
Fatigue	4.5 ± 1.4	4.4 ± 1.3	0.943
Systemic symptoms	5.3 ± 1.2	5.3 ± 1.0	0.691
Activity	5.2 ± 1.4	5.1 ± 1.4	0.668
Emotional function	5.1 ± 1.4	5.2 ± 1.3	0.574
Worry	5.0 ± 1.6	5.0 ± 1.5	0.831
**The annual rate of Platelet count, x10** ^**4**^ **/μL**	0.29 ± 2.64	0.12 ± 2.92	0.672
**ALBI score**	-0.25 ± 0.33	0.52 ± 0.48	**<0.001** *
**MELD score**	0.17 ± 1.70	2.01 ± 3.65	**0.015** *
**Child-Pugh score**	-0.42 ± 1.69	1.54 ± 2.32	**<0.001** *

SD, standard deviation; BTR, branched chain amino acid / tyrosine molar ratio; HBV, hepatitis B virus; SVR, sustained virological response; P-III-NP, procollagen type III N-terminal peptide; WFA^+^-M2BP, Wisteria floribunda agglutinin-positive mac-2 binding protein; COI, cut off index; ALBI, Albumin-Bilirubin; MELD, model for end-stage liver disease; CLDQ, chronic liver disease questionnaire

*: P value < 0.05.

**Table 4:** Univariate and multivariate logistic regression analyses in the prospective study.

	Univariable logistic regression		Multivariable logistic regression	
N = 70	Odds ratio (95% CI)	P value	Odds ratio (95% CI)	P value
**Age, years**	1.03 (0.98–1.08)	0.230		
**Gender**	1.62 (0.61–4.34)	0.335		
**Body mass index**	1.11 (0.99–1.24)	0.070		
**The etiology of liver cirrhosis**		0.931		
**Aspartate aminotransferase**	1.02 (1.00–1.03)	0.088		
**Alanine aminotransferase**	1.02 (0.99–1.04)	0.193		
**Alkaline Phosphatase**	1.00 (1.00–1.01)	0.524		
**Cholinesterase**	0.99 (0.99–1.00)	0.064		
**Albumin**	0.50 (0.21–1.17)	0.109		
**Total bilirubin**	1.11 (0.76–1.62)	0.582		
**Prothrombin time**	0.99 (0.97–1.01)	0.335		
**Ammonia**	1.01 (1.00–1.02)	0.190		
**Creatinine**	3.40 (0.76–15.2)	0.109		
**BTR**	0.99 (0.84–1.17)	0.916		
**White blood cell count**	0.88 (0.70–1.10)	0.261		
**Lymphocyte count**	0.84 (0.40–1.79)	0.654		
**Platelet count**	0.99 (0.91–1.08)	0.853		
**Control of viral hepatitis (HBV-DNA negative or SVR)**	**0.08 (0.01–0.85)**	**0.036** *		
**Hyaluronic acid**	1.00 (1.00–1.00)	0.121		
**Type IV collagen 7s**	1.03 (0.92–1.16)	0.587		
**P-III-NP**	**2.75 (1.05–7.19)**	**0.040** *		0.174
**WFA** ^**+**^ **-M2BP**	1.10 (0.98–1.23)	0.121		
**Child-Pugh score**	1.27 (0.98–1.64)	0.067		
Child Pugh grade (A/B/C)	1.70 (0.87–3.35)	0.124		
**ALBI score**	1.90 (0.85–4.28)	0.119		
ALBI grade (1/2/3)	1.44 (0.63–3.26)	0.387		
**MELD score**	**1.20 (1.03–1.40)**	**0.010** *	**1.20 (1.03–1.40)**	**0.018** *
**Total CLDQ score**	1.01 (0.66–1.55)	0.949		

CI, confidence interval; BTR, branched chain amino acid / tyrosine molar ratio; HBV, hepatitis B virus; HCV, hepatitis C virus; P-III-NP, procollagen type III N-terminal peptide; WFA^+^-M2BP, Wisteria floribunda agglutinin-positive mac-2 binding protein; ALBI, Albumin-Bilirubin; MELD, model for end-stage liver disease; CLDQ, chronic liver disease questionnaire

*: P value < 0.05.

**Fig 3:**
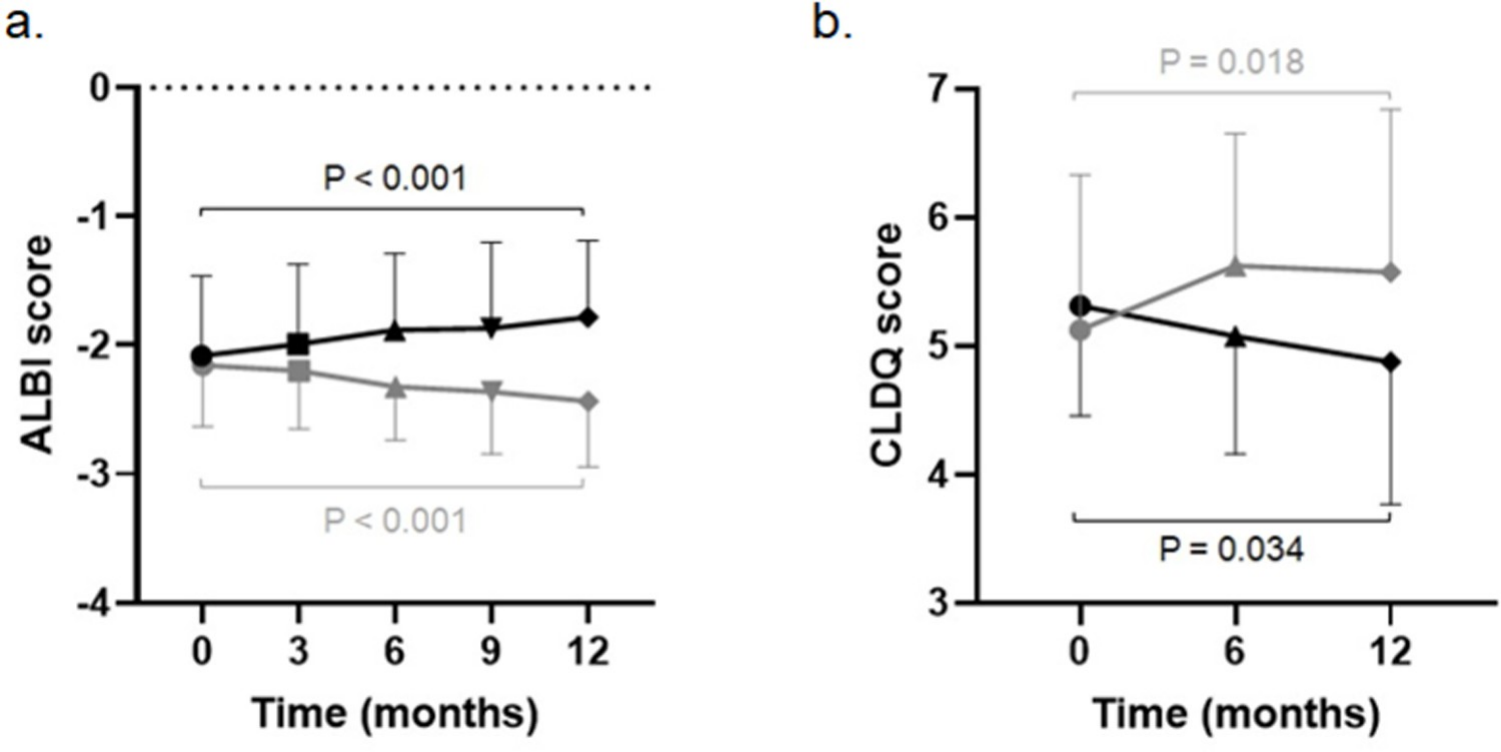
The translation of ALBI and CLDQ scores in the prospective study. In the 16 cases of the cirrhosis progression group, the median ALBI score worsened from −2.08 to −1.78 (P < 0.001), whereas in the 30 cases of the non-progression group, the median ALBI score significantly improved from −2.15 to −2.43 (P < 0.001, A). Similar to the above, the median CLDQ score worsened from 5.3 to 4.9 (P = 0.034) in the cirrhosis progression group, whereas the median CLDQ score improved from 5.1 to 5.6 (P = 0.018, B) in the non-progression group. ALBI, albumin–bilirubin; CLDQ, Chronic Liver Disease Questionnaire. Error bar, standard deviation of each data.

Furthermore, we investigated the association of the change of the CLDQ and ALBI score. Of the 60 cases observed until the end of observation period, the analysis included 46 cases who had no missing data. In the 16 cases of the cirrhosis progression group, the mean ALBI score worsened from −2.08 to −1.78 (P < 0.001), whereas in the 30 cases of the non-progression group, the mean ALBI score significantly improved from −2.15 to −2.43 (P < 0.001) (Fig 3A). Similar to the above, the mean CLDQ score in the cirrhosis progression group worsened from 5.3 to 4.9 (P = 0.034), whereas the mean CLDQ score in the non-progression group improved from 5.1 to 5.6 (P = 0.018) (Fig 3B). These results indicate that inhibiting the elevation of the ALBI score can improve not only the prognosis but also the QOL of patients.

## Discussion

Although the Child–Pugh and MELD scores have traditionally been used to evaluate liver function in patients with LC, it was reported that both scores were closely related to the grade of liver fibrosis and their prognosis [1, 21]. In our retrospective study of 89 cases, only the MELD score was significantly associated with the prognosis, whereas the ALBI and Child–Pugh scores were not, indicating that these one-point assessments were insufficient in predicting the prognosis of LC. Based on the results of the ROC analysis of the assessment of the rate of change, the ALBI score was mostly associated with the prognosis of LC. MELD score is often used for the indication of transplantation and better for patients with advanced cirrhosis [1]. On the other hand, the ALBI score is used to determine the indications for the treatment of HCC [22] and is considered to be better in compensated cirrhosis than MELD score. In our study, MELD and its change rate were more suitable for extracting cases with poor prognosis, and the change rate of ALBI were more useful the cluster frequently including compensated cirrhosis.

The ALBI score was recently developed, and its usefulness has already been reported previously [23, 24]. The ALBI score is simple and easy to calculate as it only uses two parameters. Compared with Child–Pugh score, it is also considered to be more useful in retrospective studies as the clinical symptoms do not need to be assessed. Therefore, the patients with the worsen of ALBI score should be considered a worsening of the liver function in LC. This way, prompt medical intervention can be provided to prevent the progression of LC.

In the prospective study, we analyzed the relevant parameters for the progression of LC using variable fibrosis markers and indices, and the MELD score and serum P-III-NP level were extracted as the significant parameters. Furthermore as previously reported, the acquisition of SVR in hepatitis C improves prognosis [25, 26]. Similarly, in our prospective study, where we had a small number of cases due to hepatitis C, SVR was extracted as a favorable prognostic factor. On the other hand, about 40% of the non-progression group were non-SVR cases, therefore there were cases without liver dysfunction even when the hepatitis C virus was not controlled. The MELD score is not only associated with prognosis in patients with LC but also more useful than Child–Pugh score in decompensated LC [1]. In our present study, the MELD score was significantly associated with death during the 1-year follow-up period, and a high MELD score indicated a more rapid progression to liver failure. On the other hand, the P-III-NP level was associated with the progression of LC, and a high P-III-NP level indicated a medium- to long-term poor prognosis. P-III-NP is the N-terminal peptide of procollagen type III, which is a precursor of collagen type III known as collagen in the interstitium, and has been used as a marker for liver fibrosis. However, P-III-NP is considered to represent the degree of fibrogenesis rather than fibrosis [27]. In several reports that compared the grade of fibrosis by the liver biopsies with several serum markers by ROC analysis, the AUC of the P-III-NP level was lower than that of the hyaluronic acid and FIB-4 index levels [28, 29]. Therefore, P-III-NP is not a useful marker of liver fibrosis. However, when focusing on the progression of fibrosis rather than the present fibrosis grade, P-III-NP is related to the progression and prognosis of LC. Therefore, P-III-NP may become important as a marker of control of the progression of viral hepatitis. Although the CLDQ score was affected by the patient’s background and age, the relationship between the grade of LC and the CLDQ score has been reported in several studies [30–32]. In addition, it has been reported that direct-acting antivirals for chronic hepatitis C improved the CLDQ score by achieving SVR [33, 34]. In the present study, regardless of the etiology of LC, it was shown that the QOL can be improved in patients if LC does not progress. This indicates that preventing the progression of LC is not only for the patient’s long-term prognosis but also for their good QOL.

## Conclusions

The progression of LC is associated with a poor prognosis, whereas preventing its progression improves both the prognosis and QOL. A longitudinal increase in the ALBI score is closely associated with the progression of LC. In addition, P-III-NP may be useful as a predictor of the progression of LC.

## Supporting information

S1 Table Univariate logistic regression analysis in cirrhotic patients due to hepatitis C virus. (DOCX)
