# Supplementary material for: Longitudinal increase in albumin–bilirubin score is associated with non-malignancy-related mortality and quality of life in patients with liver cirrhosis
Source: PLoS One. 2022 Feb 3;17(2):e0263464. doi: 10.1371/journal.pone.0263464 (PMC8812983; doi:10.1371/journal.pone.0263464)
Supplement: S1 Table — (DOCX) [file pone.0263464.s001.docx]

**S1 Table. Univariate logistic regression analysis in cirrhotic patients due to hepatitis C virus**

|  | Univariable logistic regression | |
| --- | --- | --- |
| **N = 19** | **Odds ratio (95% CI)** | **P value** |
| **Age, years** | 1.05 (0.96 – 1.14) | 0.310 |
| **Gender** | 5.25 (0.70 – 39.5) | 0.107 |
| **Body mass index** | 1.11 (0.92 – 1.36) | 0.277 |
| **Aspartate aminotransferase** | 1.02 (0.98 – 1.07) | 0.314 |
| **Alanine aminotransferase**  **Alkaline Phosphatase** | 1.02 (0.98 – 1.05)  0.99 (0.94 – 1.05) | 0.358  0.826 |
| **Cholinesterase** | 0.98 (0.97 – 1.00) | 0.068 |
| **Albumin** | 0.22 (0.03 – 1.58) | 0.132 |
| **Total bilirubin** | 1.35 (0.39 – 4.67) | 0.637 |
| **Prothrombin time** | 1.01 (0.97 – 1.05) | 0.606 |
| **Ammonia** | 1.00 (0.97 – 1.03) | 0.920 |
| **Creatinine**  **BTR**  **White blood cell count** | 8.94 (0.17 – 469)  0.97 (0.55 – 1.72)  1.00 (1.00 – 1.00) | 0.278  0.917  0.439 |
| **Lymphocyte count** | 1.00 (1.00 – 1.00) | 0.149 |
| **Platelet count** | 0.81 (0.60 – 1.08) | 0.157 |
| **Sustained virological response** | **0.06 (0.00 – 0.67)** | **0.023*** |
| **Hyaluronic acid** | 1.00 (1.00 – 1.00) | 0.301 |
| **Type IV collagen 7s** | 1.32 (0.91 – 1.93) | 0.142 |
| **P-III-NP** | 2.89 (0.32 – 26.5) | 0.347 |
| **WFA^+^-M2BP** | 1.23 (0.94 – 1.60) | 0.128 |
| **Child-Pugh score** | 1.25 (0.69 – 2.29) | 0.464 |
| Child Pugh grade (A/B/C) | 1.54 (0.32 – 7.48) | 0.591 |
| **ALBI score** | 3.78 (0.60 – 23.8) | 0.156 |
| ALBI grade (1/2/3) | 1.64 (0.62 – 4.35) | 0.320 |
| **MELD score** | 1.10 (0.77 – 1.57) | 0.607 |
| **Total CLDQ score** | 1.64 (0.62 – 4.35) | 0.320 |
| CI, confidence interval; BTR, branched chain amino acid / tyrosine molar ratio; P-III-NP, procollagen type III N-terminal peptide; WFA^+^-M2BP, Wisteria floribunda agglutinin-positive mac-2 binding protein; ALBI, Albumin-Bilirubin; MELD, model for end-stage liver disease; CLDQ, chronic liver disease questionnaire; *: P value < 0.05. | | |
